# Effects of canagliflozin and metformin on insulin resistance and visceral adipose tissue in people with newly-diagnosed type 2 diabetes

**DOI:** 10.1186/s12902-022-00949-0

**Published:** 2022-02-10

**Authors:** Zirui Hao, Yue Sun, Guiping Li, Yuli Shen, Yingzhen Wen, Yan Liu

**Affiliations:** 1 Department of Endocrinology, the Third People’s Hospital of Huizhou, Affiliated Huizhou Hospital of Guangzhou Medical University, Huizhou, 516000 China; 2 Department of Cardiology, The First People’s Hospital of Huizhou, Huizhou, 516003 China

**Keywords:** Diabetes mellitus, Insulin resistance, Visceral adipose tissue

## Abstract

**Background:**

The current study was to evaluate the effects of canagliflozin and metformin on insulin resistance and visceral adipose tissue in people with newly-diagnosed type 2 diabetes.

**Methods:**

This is an open-label, parallel and controlled study. Participants were divided into canagliflozin (100 mg/qd) or metformin (1000 mg/bid) groups. At baseline and after 12 weeks’ therapy, insulin resistance [Homeostatic Model Assessment of Insulin Resistance (HOMA-IR)], subcutaneous and visceral adipose tissue, fasting blood glucose (FBG), glycated hemoglobin A1c (HbA1c), C-reactive protein (CRP) and nitric oxide (NO) were evaluated and compared.

**Results:**

There was no significant between-group difference in baseline characteristics. After 12 weeks’ therapy, in canagliflozin group (*n* = 67), compared to baseline, FBG, HbA1c and HOMA-IR were decreased, accompanying with reduction of visceral adipose tissue. Compared to metformin group (*n* = 73), FBG, HbA1c and HOMA-IR were lower in canagliflozin group, accompanying with less visceral adipose tissue and lower serum CRP level and higher NO level. After multivariable regression analysis, age, visceral adipose tissue and CRP remained associated with increased insulin resistance, while canagliflozin treatment and higher NO level were associated with reduced insulin resistance. Body mass index, waist/hip ratio, CRP and HOMA-IR remained associated with increased visceral adipose tissue, while canagliflozin treatment and higher NO level were associated with reduced visceral adipose tissue. There was no difference in adverse event between these two groups.

**Conclusion:**

Canagliflozin reduces visceral adipose tissue and improves blood glucose, insulin resistance and systemic inflammation in people with newly-diagnosed type 2 diabetes.

## Background

Diabetes mellitus is a well-documented risk factor for cardiovascular and renal diseases [1–3]. In the recent decade, several clinical trials have demonstrated that sodium-glucose cotransporter-2 inhibitors (SGLT2i) have favorable effects on reducing cardiovascular and renal events in people with diabetes [4–7]. Although several theories have been proposed to explain these benefits [8, 9], the underlying mechanisms remain undetermined which deserves further elucidation [6, 9].

Among people with newly-diagnosed type 2 diabetes, results of our prior study indicated that 12 weeks’ dapagliflozin therapy was associated with significant improvement in insulin resistance and blood glucose, which might be partly attributed to the amelioration of systemic inflammation [10]. Abdominal obesity is associated with insulin resistance and chronic inflammation [11, 12] and is also highly prevalent in people with diabetes [13, 14]. Whether the benefit of SGLT2i on improving insulin resistance and blood glucose is related to the reduction of abdominal adipose tissue is unknown. Our prior study suggested that compared to baseline, the waist/hip ratio, a marker of abdominal obesity, was reduced after 12 weeks’ dapagliflozin therapy [10]. However, it was unknown whether the reduction of waist/hip ratio was due to decrease in subcutaneous or visceral adipose tissue. Prior studies have shown that compared to subcutaneous adipose tissue, visceral adipose tissue is more relevant to metabolic disorder, chronic inflammation and insulin resistance [15–18]. Therefore, assessing the changes of visceral adipose tissue with SLGT2i therapy is important for better understanding the mechanisms underlying the cardiac benefits of SLGT2i therapy. Importantly, prior studies showed that SGLT2i seemed to be useful in reducing visceral adipose tissue in people with type 2 diabetes [19, 20]. Nevertheless, there are racial/ethnic-differences in body composition as well as lifestyle and dietary pattern [21–25], and it is needed to evaluate the association between SGLT2i therapy and visceral adipose tissue changes in China’s populations with diabetes, given the high consumption of high-carbohydrate food in China.

Herein, we performed an open-label, parallel and controlled study to evaluate the effects of canagliflozin therapy on body composition, glucose control, insulin resistance and systemic inflammation in people with newly-diagnosed type 2 diabetes.

## Methods

### Study design and participant enrollment

**Fig. 1:**
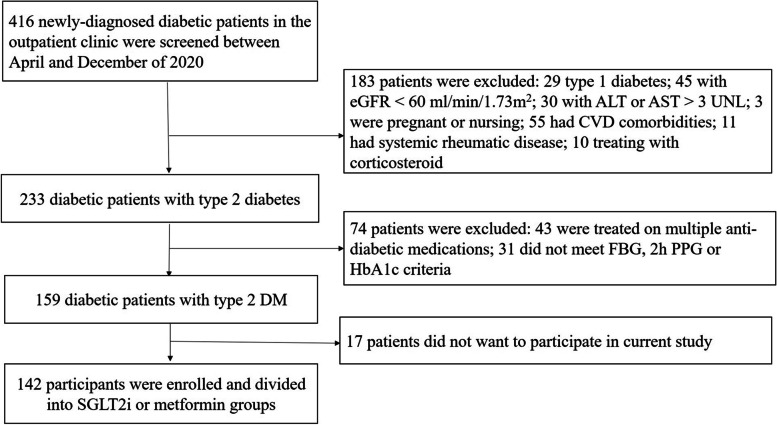
Study flowchart. eGFR, estimated glomerular filtration rate; ALT, alanine transaminase; AST, aspartate transaminase; CVD, cardiovascular disease; FBG, fasting blood glucose; PPG, post-prandial glucose; HbA1c, glycated hemoglobin A1c; SGLT2i, sodium glucose co-transporter 2 inhibitor

The current study was approved by the Institutional Review Board of the Third People’s Hospital of Huizhou and written informed consent was obtained before participants’ enrollment. This is an open-label, parallel and controlled study. All the experiment was conducted according to the Helsinki Declaration. Included criteria were as follow: ≥ 18 years old; newly-diagnosed type 2 diabetes in the last 6 months; only treated with metformin in the last 3 months and with a stable dose (500 mg/bid) in the last month; fasting blood glucose (FBG) ≥ 7 mmol/L or 2 h postprandial blood glucose ≥10.0 mmol/L for at least 2 times in the last 2 weeks, or glycated hemoglobin A1c (HbA1c) ≥ 7.5% in the last 3 months. Excluded criteria were as follow: type 1 diabetes, estimated glomerular filtration rate (eGFR) < 60 ml/min/1.73m^2^, baseline alanine transaminase (ALT) or aspartate transaminase (AST) level ≥ 3-fold of upper normal limit, pregnant or nursing women, acute coronary syndrome, congestive heart failure, ischemic stroke or cerebrovascular hemorrhage in the last 6 months, malignancy disease, systemic rheumatic disease or treated with glucocorticoid or anti-inflammatory drugs. Based on the last digit number of their telephone number, participant was divided into the active group (canagliflozin 100 mg/qd) with odd number or into the control group (metformin 1000 mg/bid) with even number (Fig. 1).

### Data collection

Standardized questionnaire was used to collect data on demographics (age and gender). Measurements of anthropometrics (weight, height, and waist and hip circumference) were performed by trained staff using standardized protocol. Obesity was defined as body mass index (BMI) ≥ 28 kg/m^2^ [26], and waist/hip ratio > 0.85 in women and > 0.90 in men was defined as abdominal obesity [27]. Subcutaneous and visceral adipose tissue were determined using computed tomography cross-sectional images of the abdomen at the umbilicus level. In brief, contiguous slices centered on the umbilicus level were obtained for quantifying the volume of subcutaneous and visceral adipose tissue. We traced the abdominal muscular wall manually. The fat volumes in different compartments were determined using semiautomatic segmentation technique, and the volume of subcutaneous and visceral adipose was calculated separately using the Slim Vision (CYBERNET SYSTEMS CO., LTD., Tokyo, Japan). The abdominal computed tomography images were carried out by an experienced radiologist, who was blinded to the clinical characteristics and group signing of the participants. Comorbid conditions, including cigarette smoking, hypertension, dyslipidemia, prevalent cardiovascular disease (CVD), and current medication used were collected by two independent investigators using standardized questionnaire. In brief, the status of physical activity was determined if the people who had regular physical exercise (such as jogging or running) at least 30 min/day for 5 days per week in the last month [28]. Otherwise, the people were considered as physical inactivity.

### Laboratory examination

At baseline and after 12 weeks’ treatment, fasting venous blood were drawn for laboratory examination. In brief, FBG, HbA1c, serum levels of creatinine, ALT and AST, insulin, C-reactive protein (CRP) and nitric oxide (NO) were measured in the core lab of our hospital using the standardized method as our prior report [10]. Homeostatic Model Assessment of Insulin Resistance (HOMA-IR) was calculated as fasting insulin (mIU/mL) * fasting glucose (mmol/L) / 405 as described previously [29].

### Adverse events

Adverse events related to therapy included rash and allergy, hypoglycemia, diarrhea, abdominal pain, liver function impairment, urinary tract infection, lactic acidosis and diabetic ketoacidosis. All participants were informed the potential adverse events before enrollment and were asked to return to the clinic to confirm the potential adverse events. All these adverse events were adjudicated by two independent physicians.

### Statistical analysis

Based on our prior results [10], 70 participants in each group would have 90% statistical power to detect the between-group difference in FBG, HOMA-IR and CRP, with a two-sided *P*-value < 0.05. Therefore, we planned to enroll 140 participants for the current study. Continuous variables were presented as mean ± standard deviation (SD) and compared by the student *t* test; and categorical variables were presented by number (proportion) and compared by the chi-square or Fisher exact test. Univariate regression analysis was performed to evaluate factors associated with increased insulin resistance and visceral adipose tissue, and factors with a *P*-value < 0.1 were entered into multivariable regression analysis. Odds ratio (OR) and 95% confidence interval (CI) was reported. All the statistical analysis was performed using SPSS 24.0 (SPSS Inc., Chicago, IL). Two-sided *P*-value < 0.05 was considered statistically significant.

## Results

From April to December of 2020, 142 people with newly diagnosed type 2 diabetes were enrolled, and 69 were in the active group and 73 were in the control group. The mean age of all participants was 56.2 ± 11.8 years, females accounted for 47.2% (*n* = 67), and the mean duration of diabetes was 5.3 ± 0.5 months.

### Comparisons of baseline characteristics

The mean age in both groups were 57.3 ± 9.8 and 56.0 ± 8.5 years, and females accounted for 47.8 and 46.6%, respectively (Table 1). The mean duration of diabetes was 5.2 ± 0.6 and 5.3 ± 0.6 months, respectively. The prevalence of general obesity, abdominal obesity and comorbid conditions were similar between these two groups, as was the medications used at baseline.

**Table 1:** Comparisons of baseline characteristics

Variables	Active group (*n* = 69)	Control group (*n* = 73)
Age (years)	57.3 ± 9.8	56.0 ± 8.5
Female, n (%)	33 (47.8)	34 (46.6)
General obesity, n (%)	39 (56.5)	40 (54.8)
Abdominal obesity, n (%)	30 (43.5)	32 (43.8)
Current smoking, n (%)	29 (42.0)	30 (41.1)
Duration of diabetes (months)	5.2 ± 0.6	5.3 ± 0.6
Physical inactivity, n (%)	47 (68.1)	48 (65.8)
Hypertension, n (%)	50 (72.4)	52 (71.2)
Dyslipidemia, n (%)	45 (65.2)	47 (64.4)
Prior CVD history, n (%)	10 (14.5)	8 (11.0)
Aspirin, n (%)	29 (42.0)	29 (39.7)
Statins, n (%)	27 (39.1)	26 (35.6)
Anti-hypertensive medications, n (%)	42 (60.9)	43 (58.9)

*CVD* cardiovascular disease

### Comparisons of selected parameter at baseline and at 12 weeks

At baseline, there were no between-group differences in the laboratory indices, BMI, waist/hip ratio, subcutaneous and visceral adipose tissue (Table 2). After 12 weeks, compared to baseline, FBG, HbA1c, HOMA-IR and visceral adipose tissue were decreased, and systemic inflammation and endothelial function were improved in the active group. In addition, compared to the control group, FBG, HbA1c, HOMA-IR, visceral adipose tissue and serum CRP level were lower, and serum NO level was higher in the active group.

**Table 2:** Changes and comparisons of selected parameters (follow-up minus baseline)

Variables	Baseline	12 weeks	Change
FBG (mmol/L)			
Active group	7.7 ± 0.5	6.5 ± 0.5*	−1.2 ± 0.4
Control group	7.6 ± 0.6	7.3 ± 0.6^#^	−0.3 ± 0.2
HbA1c (%)			
Active group	7.9 ± 0.4	7.1 ± 0.5*	−0.8 ± 0.4
Control group	8.0 ± 0.4	7.8 ± 0.6^#^	−0.2 ± 0.2
HOMA-IR			
Active group	2.5 ± 0.4	1.7 ± 0.5*	−0.8 ± 0.4
Control group	2.4 ± 0.4	2.1 ± 0.4^#^	−0.3 ± 0.2
BMI, kg/m^2^			
Active group	26.4 ± 5.0	25.9 ± 4.4	−0.5 ± 0.4
Control group	26.3 ± 4.8	26.1 ± 4.1	−0.2 ± 0.1
Waist/hip ratio			
Active group	0.83 ± 0.11	0.80 ± 0.10	−0.03 ± 0.01
Control group	0.84 ± 0.10	0.83 ± 0.12	−0.01 ± 0.01
Subcutaneous adipose tissue (cm^2^)			
Active group	206.7 ± 50.3	203.2 ± 47.1	−3.5 ± 1.0
Control group	204.2 ± 48.8	201.8 ± 44.3	−2.4 ± 0.8
Visceral adipose tissue (cm^2^)			
Active group	154.6 ± 41.8	146.8 ± 40.4*	−7.6 ± 2.3
Control group	152.9 ± 39.2	150.3 ± 38.3^#^	−2.6 ± 0.9
ALT (U/L)			
Active group	38 ± 12	35 ± 15	−3 ± 2
Control group	36 ± 14	34 ± 14	−2 ± 2
AST (U/L)			
Active group	36 ± 13	33 ± 11	−3 ± 1
Control group	37 ± 13	34 ± 12	−3 ± 2
Creatinine (μmol/L)			
Active group	1.1 ± 0.3	1.0 ± 0.4	−0.1 ± 0.1
Control group	1.1 ± 0.4	1.0 ± 0.3	−0.1 ± 0.1
C-reactive protein (mg/L)			
Active group	5.8 ± 1.2	3.3 ± 0.8*	−1.5 ± 0.6
Control group	5.9 ± 1.1	4.6 ± 0.9^#^	−1.3 ± 0.5
Nitric oxide (μmol/L)			
Active group	46.8 ± 9.6	52.4 ± 11.5*	5.6 ± 0.8
Control group	45.4 ± 8.8	48.7 ± 9.6^#^	3.3 ± 0.6

*FBG* fasting blood glucose, *HbA1c* glycated hemoglobin A1c, *HOMA-IR* homeostatic model assessment of insulin resistance, *BMI* body mass index, *ALT* alanine transaminase, *AST* aspartate transaminase; Change = variable value at baseline minus that at 12 weeks; * *P* < 0.05 versus baseline in the active group; ^#^ *P* < 0.05 versus the active group at 12 weeks

### Factors associated with increased insulin resistance and visceral adipose tissue

In the univariate regression analysis (Table 3), factors associated with insulin resistance included age, BMI, waist/hip ratio, visceral adipose tissue, canagliflozin treatment, CRP and NO. After multivariable regression analysis, age, visceral adipose tissue, and CRP remained associated with increased insulin resistance, while canagliflozin treatment and higher serum NO level was associated with reduced insulin resistance.

**Table 3:** Factors associated with increased insulin resistance

	OR and 95% CI		OR and 95% CI	
	Univariate analysis	*P* value	Multivariable analysis	*P* value
Age (per 10 years increase)	1.26 (1.14–1.51)	< 0.001	1.09 (1.02–1.26)	0.04
Gender (female vs male)	1.08 (0.96–1.22)	0.25	N/A	
BMI (per 5 kg/m^2^ increase)	1.23 (1.11–1.36)	< 0.001	1.05 (0.91–1.13)	0.19
Waist/hip ratio (per 0.1 increase)	1.36 (1.24–1.58)	< 0.001	1.17 (0.98–1.26)	0.08
Subcutaneous adipose tissue (per 5 cm^2^ increase)	1.08 (0.96–1.23)	0.08	1.02 (0.90–1.17)	0.26
Visceral adipose tissue (per 5 cm^2^ increase)	1.50 (1.39–1.82)	< 0.001	1.26 (1.11–1.63)	0.008
Smoking (yes vs no)	1.06 (0.87–1.10)	0.39	N/A	
Physical inactivity (yes vs no)	1.12 (1.03–1.25)	0.03	1.04 (0.90–1.09)	0.35
Hypertension (yes vs no)	1.05 (0.92–1.14)	0.34	N/A	
Dyslipidemia (yes vs no)	1.13 (0.95–1.30)	0.09	1.05 (0.89–1.07)	0.41
Prior CVD history (yes vs no)	1.05 (0.86–1.05)	0.42	N/A	
Statin (yes vs no)	0.90 (0.82–1.03)	0.11	N/A	
Canagliflozin vs Metformin	0.83 (0.72–0.90)	0.009	0.90 (0.83–0.97)	0.02
CRP (per 1 mg/L increase)	1.21 (1.09–1.47)	< 0.001	1.08 (1.01–1.25)	0.04
NO (per 5 μmol/L increase)	0.80 (0.67–0.91)	< 0.001	0.85 (0.74–0.97)	0.01

*OR* odds ratio, *CI* confidence interval, *BMI* body mass index, *CVD* cardiovascular disease, *CRP* C-reactive protein, *N/A* no applicable

After multivariable regression analysis, BMI, waist/hip ratio, CRP and HOMA-IR remained associated with increased visceral adipose tissue, while canagliflozin treatment and higher serum NO level was associated with reduced visceral adipose tissue (Table 4).

**Table 4:** Factors associated with increased visceral adipose tissue

	OR and 95% CI		OR and 95% CI	
	Univariate analysis	*P* value	Multivariable analysis	*P* value
Age (per 10 years increase)	1.11 (10.1–1.20)	0.04	1.04 (0.92–1.13)	0.28
Gender (female vs male)	0.93 (0.88–1.04)	0.15	N/A	
BMI (per 5 kg/m^2^ increase)	1.20 (1.05–1.36)	0.02	1.13 (1.02–1.27)	0.04
Waist/hip ratio (per 0.1 increase)	1.33 (1.21–1.54)	< 0.001	1.25 (1.11–1.46)	0.007
Subcutaneous adipose tissue (per 5 cm^2^ increase)	1.26 (1.17–1.42)	< 0.001	1.08 (0.99–1.20)	0.06
Smoking (yes vs no)	1.05 (0.92–1.10)	0.43	N/A	
Physical inactivity (yes vs no)	1.17 (1.06–1.32)	0.03	1.06 (0.95–1.10)	0.37
Hypertension (yes vs no)	1.04 (0.95–1.16)	0.55	N/A	
Dyslipidemia (yes vs no)	1.15 (1.04–1.32)	0.04	1.05 (0.96–1.14)	0.10
Prior CVD history (yes vs no)	1.06 (0.90–1.13)	0.31	N/A	
Statin (yes vs no)	0.92 (0.85–1.01)	0.07	0.94 (0.88–1.05)	0.19
Canagliflozin vs Metformin	0.86 (0.79–0.91)	0.008	0.90 (0.84–0.98)	0.04
CRP (per 1 mg/L increase)	1.27 (1.10–1.52)	< 0.001	1.10 (1.01–1.23)	0.03
NO (per 5 μmol/L increase)	0.78 (0.66–0.89)	< 0.001	0.83 (0.76–0.94)	0.01
HOMA-IR (per 0.5 increase)	1.40 (1.28–1.85)	< 0.001	1.29 (1.17–1.60)	0.004

*OR* odds ratio, *CI* confidence interval, *BMI* body mass index, *CVD* cardiovascular disease, *CRP* C-reactive protein, *HOMA-IR* homeostatic model assessment of insulin resistance, *N/A* no applicable

### Comparisons of adverse events

The overall rate of adverse events was low in both groups (8.7% vs 6.8%). There was no difference in individual adverse event between the active and control groups. Participants with canagliflozin treatment had a numerically higher rate of genital mycotic infection (Table 5), and all these cases occurred in female participants. There was no participant developed lactic acidosis or diabetic ketoacidosis in both groups.

**Table 5:** Comparisons of adverse events

	Active group (*n* = 69)	Control group (*n* = 73)	*P*-value
Rash/allergy, n (%)	1 (1.4)	0	0.89
Hypoglycemia, n (%)	1 (1.4)	1 (1.4)	0.72
Diarrhea, n (%)	0	2 (2.7)	0.93
Abdominal pain, n (%)	1 (1.4)	1 (1.4)	0.99
Liver function impairment, n (%)	0	0	N/A
Genital mycotic infection, n (%)	3 (4.3)	1 (1.4)	0.15
Lactic acidosis, n (%)	0	0	N/A
Diabetic ketoacidosis, n (%)	0	0	N/A
Overall, n (%)	6 (8.7)	5 (6.8)	0.50

*N/A* no applicable

## Discussion

The current study was aimed to evaluate the effects of canagliflozin treatment on body composition and insulin resistance in people with newly-diagnosed type 2 diabetes. There are three important findings. First, compared to metformin, canagliflozin therapy had a better improvement in blood glucose. Second, canagliflozin therapy was associated with insulin resistance improvement and visceral adipose tissue reduction. Third, the rates of adverse event were similar between these two groups.

Several large randomized clinical trials have demonstrated that SGLT2i therapy is beneficial for reducing cardiovascular and renal events in people with diabetes [4–7]. However, the underlying mechanisms are not fully understood. Improvement in insulin resistance, amelioration of systemic inflammation and improvement in endothelial function have been proposed to explain the benefits of SGLT2i therapy [3, 30, 31]. Abdominal obesity is associated with cardiometabolic disorders, systemic inflammation and endothelial dysfunction [17, 32–34]. Therefore, to elucidate whether SGLT2i therapy is associated with visceral adipose tissue reduction is important to further understand the cardio-renal benefits of SGLT2i therapy.

Results of our prior study suggests that after 12 weeks’ dapagliflozin therapy, there was no significant change in the waist/hip ratio when compared to baseline [10]. Waist/hip ratio is a marker of abdominal obesity while it cannot differentiate between subcutaneous and visceral adipose tissue [11, 35–37]. Compared to subcutaneous adipose tissue, visceral adipose tissue was more atherogenic and more relevant to cardiometabolic disorder [11, 35–37]. Accordingly, we compared the changes of subcutaneous and visceral adipose tissue after 12 weeks’ canagliflozin therapy. Consistent with our prior report [10], there was no significant change in waist/hip ratio between baseline and after 12 weeks’ therapy. Nevertheless, there was significant change in visceral adipose tissue when compared to baseline. In addition, when compared to the control group, the volume of visceral adipose tissue was also lower. Compared to baseline, FBG, HbA1c, HOMA-IR, CRP and NO were all improved after 12 weeks’ canagliflozin therapy. Notably, NO is a marker of endothelial function, and the improvement in NO suggests that canagliflozin had beneficial effect on improving endothelial function, which might be partially ascribed to the improvement in insulin resistance. In addition, improvement in endothelial function could also be an indicator of improvement in diabetes mellitus, which is an important determinant of endothelial function. Compared to the control group, these improvements were greater in the canagliflozin group. After adjusting for multiple covariates, canagliflozin therapy was associated with visceral adipose tissue reduction. Importantly, prior studies also suggest that on top of sitagliptin, ipragliflozin therapy had greater effects on reducing visceral fat reduction and improving metabolic dysfunction when compared to metformin [38, 39]. Both prior and our current results demonstrate that regardless of the background antidiabetic therapy, SGLT2i appeared to be better than metformin in reducing visceral adipose tissue. While there was no significant change in subcutaneous adipose tissue, which was different from prior report [19]. These differences might be due to ethnic differences in body composition, lifestyle or diet pattern, specific SGLT2i used, and duration of SGLT2i used. Notwithstanding, our current findings suggest that the beneficial effects of canagliflozin therapy might be ascribed to the reduction of visceral adipose tissue rather than subcutaneous adipose tissue. Further studies are needed to corroborate the current findings and illustrate the mechanisms underlying the differential effects of canagliflozin on subcutaneous and visceral adipose tissue.

We further evaluated the relationship between canagliflozin therapy and insulin resistance. Similar to dapagliflozin therapy, canagliflozin therapy was associated with improvement in insulin resistance, which might be attributed to the reduction of visceral adipose tissue. There was no significant relationship between subcutaneous adipose tissue and insulin resistance, further supporting the importance of visceral adipose tissue in the development of insulin resistance [11, 40]. In addition, these results also indirectly indicate that visceral adipose tissue might be the therapeutic target of canagliflozin therapy. Further studies are needed to corroborate the current findings. Considering the substantial cardiovascular and renal benefits of SGLT2i therapy, it has been discussed that whether SLGT2i can be used as the first-line therapy for people with type 2 diabetes [41]. The 2020 China Diabetes Society Guideline still recommends metformin as the first-line therapy for people with diabetes in the context of without prevalent cardiovascular and renal disease [42]. Results of the current study suggested that SGLT2i, such as canagliflozin, might be used as the first-line therapy due to its better performance in improving insulin resistance and blood glucose.

After 12 weeks’ therapy, there was no significant difference in the rate of adverse events between the canagliflozin and metformin groups, supporting the safety profile of canagliflozin therapy. In addition, it also suggests that in people with newly-diagnosed type 2 diabetes, SGLT2i therapy might confer additional benefits for the management of diabetes and cardiometabolic disorders.

### Limitations

There are some limitations of the current study. First, this was a single center and open-label study. Therefore, no causal relationship could be drawn, and potential selection bias might exist due to the non-randomized design. Second, participants recruited in the current study were newly-diagnosed type 2 diabetes and whether these findings can be extrapolated to individuals with longstanding diabetes is unknown. Third, this was a short-term study and whether the beneficial effects of canagliflozin therapy can be extended to long-term follow-up is unknown. Further studies with long-term follow-up are needed. Fourth, all participants in the current study are Chinese and considering the ethnic differences in body composition, whether these findings can be extrapolated to other ethnic groups were unknown. Fifth, it is well documented that physical activity is important for insulin resistance improvement. Nevertheless, in the current study, we did not capture detailed information on physical activity, which prohibited us to obtain better insight on the effects of physical activity on insulin resistance in people with diabetes. Further studies are needed to better understand whether physical activity and canagliflozin therapy could have additive effects on insulin resistance. Sixth, the current study used the last digit number of telephone to simulate randomized assignment which could not represent truly randomization. Further randomized clinical trials are needed to confirm our preliminary results. Last but not the least, the dose of metformin used in the current study was 1000 mg/bid which might be insufficient to control blood glucose. Further studies are needed to assess whether high dose of metformin could provide comparable effect on glucose control when compared to canagliflozin.

## Conclusion

In conclusion, the current study shows that compared to metformin therapy, canagliflozin therapy reduces visceral adipose tissue, and improves blood glucose, insulin resistance and systemic inflammation in people with newly-diagnosed type 2 diabetes.

## Data Availability

The datasets used and/or analysed during the current study available from the corresponding author on reasonable request.

## Abbreviations

HOMA-IR: Homeostatic Model Assessment of Insulin Resistance
FBG: Fasting blood glucose
HbA1c: Glycated hemoglobin A1c
CRP: C-reactive protein
NO: Nitric oxide
SGLT2i: Sodium-glucose cotransporter-2 inhibitors
eGFR: Estimated glomerular filtration rate
ALT: Alanine transaminase
AST: Aspartate transaminase
BMI: Body mass index
SD: Standard deviation
OR: Odds ratio
CI: Confidence interval
